# Hepatic Epstein-Barr Virus-Associated Smooth Muscle Tumor in a Young Woman With Congenital HIV

**DOI:** 10.7759/cureus.95043

**Published:** 2025-10-21

**Authors:** Mariam Hafez, Ahmed Ebeid, Alisa Dewald, Abdelrhman Refaey, Reid Schalet, Mamoun Younes, Ameer Abutaleb

**Affiliations:** 1 Transplant Hepatology, George Washington University School of Medicine and Health Sciences, Washington, DC, USA; 2 Internal Medicine Department, Howard University Hospital, Washington, DC, USA; 3 Internal Medicine, Holy Name Medical Center, Teaneck, USA; 4 Internal Medicine, George Washington University School of Medicine and Health Sciences, Washington, DC, USA; 5 Department of Pathology, George Washington University School of Medicine and Health Sciences, Washington, DC, USA

**Keywords:** biopsy, congenital immunodeficiency, epstein-barr virus, leiomyoma, smooth muscle tumor

## Abstract

Epstein-Barr virus-associated smooth muscle tumor (EBV-SMT) is a rare condition involving smooth muscle tumors in various organs, often in the context of immunodeficiency. Hepatic EBV-SMT usually presents with non-specific symptoms such as abdominal pain and weight loss. We present a case of a 25-year-old woman who presented with multiple liver masses in the setting of congenital human immunodeficiency virus (HIV). Initially, those lesions were suspected to represent metastatic tumors, but the biopsy showed smooth muscle proliferation suggestive of a smooth muscle tumor. Diagnosis of EBV-SMT was confirmed through in situ hybridization for EBV RNA. This case underscores the diagnostic challenges and pathogenesis of EBV-SMT.

## Introduction

Epstein-Barr virus-associated smooth muscle tumor (EBV-SMT) is an exceptionally rare neoplastic condition characterized by the development of SMTs in various organs, often associated with EBV infection [1]. This pathology is notably infrequent, and its clinical presentation and manifestations can vary widely depending on where the tumor is located. Most cases of EBV-SMT are located in the liver, but it could affect other organs such as the lungs, the central nervous system, and the gastrointestinal tract [2,3]. Although tumors may have a multifocal pattern, this is not due to metastases but rather multiple infection events [4]. EBV-SMT of the liver usually presents with non-specific symptoms such as fever, weight loss, headache, GI upset, hematuria, and shortness of breath [5]. Typically, EBV-SMTs occur in immunocompromised patients through several proposed mechanisms involving latent membrane proteins (LMPs) and Epstein-Barr virus nuclear antigens (EBNAs) [6] and are typically mediated by epigenetic modifications, chromosome instability, and mutations in DNA mismatch repair [7]. Due to the rarity of this condition, diagnostic challenges may arise, particularly because it is EBV-mediated and demands meticulous examination of biopsy specimens.

## Case presentation

A 25-year-old woman with a known history of congenital human immunodeficiency virus (HIV) (non-adherent to Biktarvy), end-stage renal disease (ESRD), and multiple liver lesions presented as a transfer from an outside hospital after having a new onset of generalized tonic-clonic seizures. On admission, the patient reported dizziness, fatigue, diffuse abdominal, back, and bilateral leg pains with symmetric weakness. Physical examination revealed diffuse abdominal tenderness with genital skin lesions. Laboratory workup showed elevated levels of alkaline phosphatase, aspartate transaminase (AST), and alanine transaminase (ALT) with a normal total protein level. Her HIV viral load was undetectable, but her CD4 count was markedly low (Table 1). The MRI brain was normal. EEG did not capture any events. Psychogenic nonepileptic seizures were suspected, and psychiatry was consulted for further evaluation. 

**Table 1:** Laboratory values ordered during the patient's inpatient hospitalization.

Lab tests	Lab values (ordered day 0 upon admission)	Lab values (ordered day 13)	Reference range
WBC	4.18 million cells/mcL	6.56	4.80-10.80 million cells/mcL
RBC	2.51↓ million cells/mcL	2.57 ↓	4.20-5.40 million cells/mcL
Hb	7.8 ↓ gm/dL	8.30 ↓	12-16 gm/dl
Total Protein	5.2 gm/dL	8.60	6-8 gm/dL
Alk Phos	908↑ units/L	1156 ↑	40-125 units/L
AST	83 ↑ units/L	54 ↑	15-50 units/L
ALT	59↑ units/L	68 ↑	10-45 units/L
EBV DNA Qnt	192 ↑ IU/mL	-	Not detected
EBV Capsin Antigen (VCA) IgG Ab	>160 ↑ U/mL	-	Negative (uninfected)
EBV Nuclear Antigen	525 ↑ U/mL	-	Negative (uninfected)
CMV DNA Qnt	<34.5 IU/mL	-	Not detected (<34.50 IU/mL)
HIV-RNA Qnt	Not detected	-	Not detected
CD4%	2% ↓	-	30-60%
CD4 Absolute	22 ↓ Cells/mcL	-	491–1381 cells/μL
Ferritin	1,950 ↑ ng/mL	1560 ↑	6-130 ng/mL
Alpha-fetoprotein	-	8.85 ↑ ng/mL	0-7.22 ng/mL
CA 125	-	<21	0-35 U/mL
CA 19-9	-	<14	0-37 U/mL
CEA	-	6.85 ↑ ng/mL	0-5.90 ng/mL
Ceruloplasmin	-	8.80 ↑ mg/dL	19-39 mg/dL
Actin (smooth muscle) Ab	-	28 ↑ units	0-19 units

**Figure 1:**
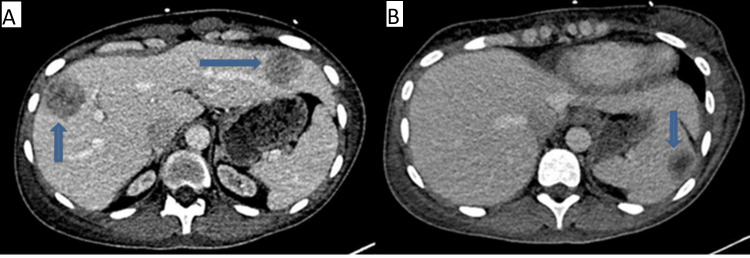
A. CT Abdomen revealed multiple solid hypoenhancing hepatic lesions with the largest measuring 3.7x3.5 cm in the right hepatic lobe and B. hypoenhancing splenic lesion measuring 2.7x2.1 cm. (blue arrows)

A CT abdomen with contrast was done and revealed multiple hepatic lesions involving both the right and left lobes, as well as a splenic lesion (Figure 1). 

**Figure 2:**
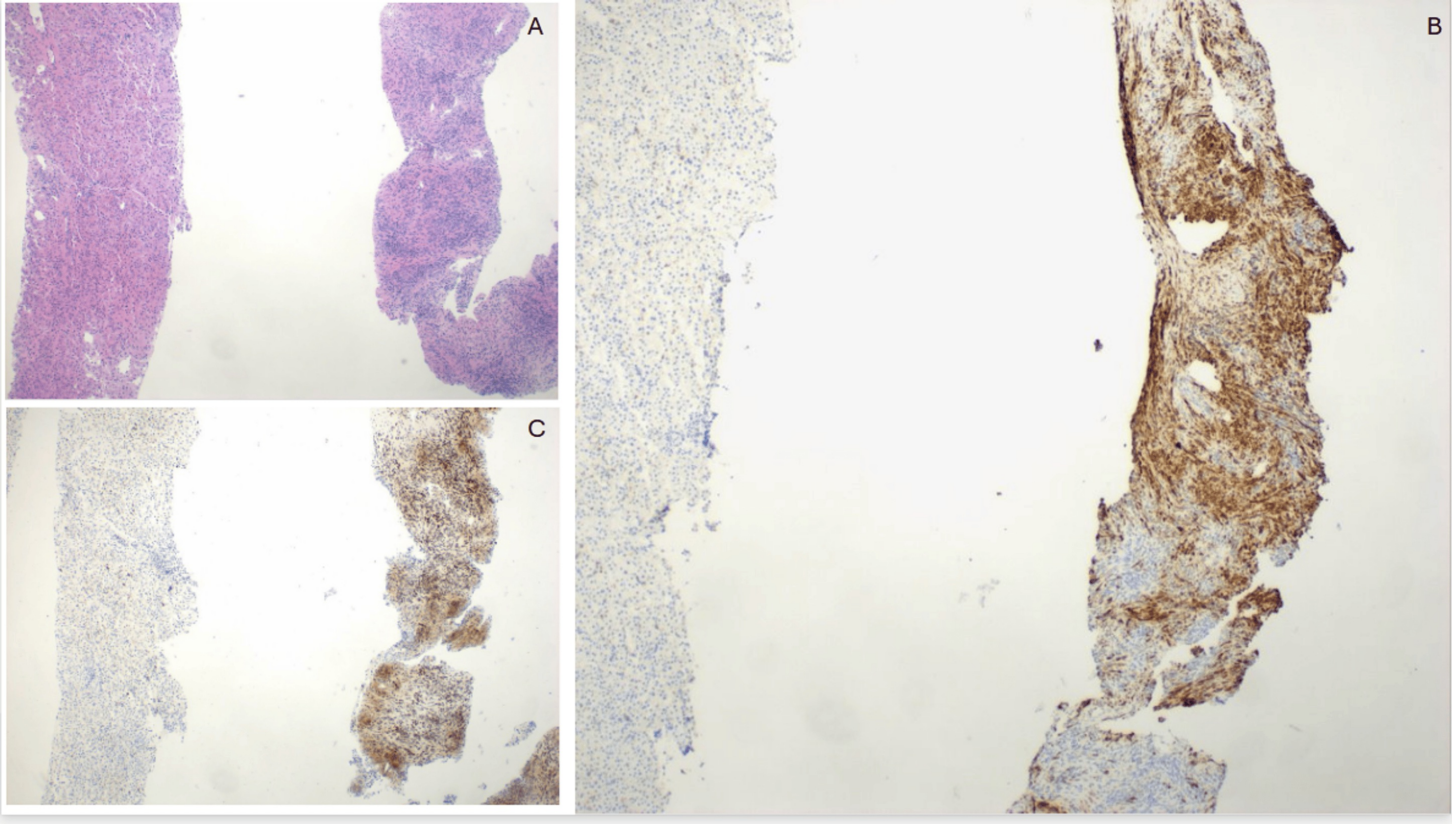
Liver biopsy showing a benign-appearing spindle cell neoplasm. (A) Hematoxylin and eosin (H&E) stain demonstrating intersecting fascicles of spindle-shaped cells with eosinophilic cytoplasm and elongated, blunt-ended nuclei (low power). (B) Immunohistochemistry showing diffuse, strong cytoplasmic positivity for smooth muscle actin (SMA). (C) Immunohistochemistry showing diffuse cytoplasmic positivity for desmin. Findings are consistent with a smooth muscle neoplasm.

CT-guided biopsy of one of the right lobe hepatic lesions demonstrated smooth muscle proliferation suggestive of leiomyoma (Figure 2). Given the patient’s immunocompromised state and persistent diagnostic uncertainty, a second biopsy was performed to further evaluate concurrent pathology. It revealed siderosis and mild portal and periportal fibrosis.

Given the patient’s history and the benign-appearing liver biopsy results, disseminated peritoneal leiomyomatosis was initially considered. Additional laboratory tests were ordered to better investigate the definite diagnosis and to better assess the abnormal liver enzymes. 

A retrospective review of the patient's medical records revealed a diagnosis of EBV-SMT that had been established two years earlier, of which the patient was previously unaware. Consequently, tumor markers were ordered, and her case was referred to the tumor board for multidisciplinary discussion. Laboratory studies showed elevated EBV DNA levels, EBV capsid antigen (VCA) IgG Ab, and EBV nuclear antigen (Table 1). 

Comparing the current imaging and biopsy findings with the previous ones raised concern for recurrent EBV-SMT. Using in situ hybridization for EBV RNA on the recent liver biopsy at our hospital, returned positive, confirming recurrence. The patient was counseled about her diagnosis and restarted on antiretroviral therapy (Biktarvy plus dapsone) and scheduled for follow-up at the cancer center. 

## Discussion

EBV-SMT arises from the oncogenic potential of the EBV, a ubiquitous herpes virus infecting a large proportion of the human population worldwide. The virus can persist latently in B lymphocytes and epithelial cells, and under certain conditions, it can lead to the transformation of smooth muscle cells, culminating in tumor formation [8].

Clinical presentation of EBV-SMTs is nonspecific, with symptoms varying based on tumor size and location [1]. Presentation may also vary by the cause of immunosuppression, with transplant-associated EBV-SMT presenting on average about 50 months after surgery versus HIV-associated EBV-SMT presenting up to 18 years after diagnosis [4]. This correlates with our case being diagnosed with congenital HIV. Risk factors related to transplant-associated EBV-SMT are not well studied, but it is hypothesized that pre-transplant seronegativity, along with EBV infection with high viral load, may increase the risk for post-transplant EBV-SMT [9]. As the patient presented in this case report did not have a history of organ transplant, it is important to note the increased risk of EBV-SMT in all immunosuppressed patients. 

Diagnosis cannot be made based on imaging alone and must be confirmed through a biopsy with in-situ hybridization for Epstein-Barr encoded RNA (EBER) and immunohistochemistry [10]. EBV-SMTs are characterized histologically by smooth muscle cells with atypical nuclei and intra-tumoral T lymphocytes. Grossly, tumors can reach up to 21 cm and are typically firm, hemorrhagic, soft tissue masses that may be well circumscribed [11]. Our case presents a patient with a liver biopsy consistent with leiomyoma with positive in situ hybridization for EBV mRNA. Leiomyomas are similar to EBV-SMTs histologically but are negative for in situ hybridization [12]. Differential diagnosis also includes leiomyosarcoma, which can be distinguished histologically from EBV-SMT and leiomyoma by nuclear atypia and mitotic activity [12]. 

The treatment of EBV-SMT focuses on mitigating immunosuppression by discontinuing immunosuppressive medications, with surgical treatment for respectable tumors [13]. Recent literature also suggests that for those with HIV-associated EBV-SMT, improvement in CD4+ count can lead to a decrease in tumor size without surgery [14]. One case even reported complete metabolic remission after antiretroviral treatment alone [15]. Although higher CD4+ count may be linked to lower mortality, the relationship between tumor features on histopathology and tumor behavior still remains unclear [16,17]. Among all causes of EBV-SMT, HIV-associated EBV-SMT bears the worst survival prognosis [16]. 

In this report, we present the case of a 25-year-old female admitted for nonspecific seizure activity who was found to have EBV-SMT. However, a lack of adequate patient communication and poor linkage of care led to delayed treatment.

Although rare, these tumors are slow-growing, with a one-year survival rate of 50-76% [3]. Given this, there is a serious potential for morbidity if the disease is not diagnosed and treated promptly. This case underlines the importance of timely and appropriate diagnosis and the need for vigilance in immunocompromised patients to avoid delaying treatment and improve outcomes. 

## Conclusions

EBV-SMTs in immunocompromised individuals are uncommon and often difficult to diagnose due to their nonspecific clinical presentations and radiologic findings, typically necessitating biopsy and EBV-specific in situ hybridization for definitive confirmation. These tumors may recur, particularly in patients with persistently low CD4 counts or inconsistent adherence to immune-restorative therapy. They can arise in multiple or atypical anatomical locations. Restoration of immune function through sustained antiretroviral therapy in HIV-positive patients or by reducing immunosuppression in transplant recipients is central to improving prognosis and may result in tumor regression. Surgical resection remains an option for localized or symptomatic cases. Physicians should maintain a strong clinical suspicion for EBV-SMTs in immunocompromised patients with unexplained masses, as timely diagnosis and coordinated multidisciplinary care are vital in minimizing morbidity and enhancing survival.
